# Supplementary material for: Spatial decoupling of bromide-mediated process boosts propylene oxide electrosynthesis
Source: Nat Commun. 2024 Apr 29;15:3646. doi: 10.1038/s41467-024-48070-1 (PMC11059342; doi:10.1038/s41467-024-48070-1)
Supplement: Supplementary file 1 — Supplementary Information [file 41467_2024_48070_MOESM1_ESM.pdf]

Supplementary Information for

Spatial decoupling of bromide-mediated process boosts propylene oxide electrosynthesis

Mingfang Chi[1,†], Jingwen Ke[1,†], Yan Liu[1,†], Miaojin Wei[1], Hongliang Li[1], Jiankang Zhao[1], Yuxuan Zhou[1], Zhenhua Gu[1], Zhigang Geng[1,*], Jie Zeng[1,2,3,4,5,*]

[1]Hefei National Research Center for Physical Sciences at the Microscale, University of Science and Technology of China, Hefei, Anhui 230026, P. R. China.

[2]CAS Key Laboratory of Strongly-Coupled Quantum Matter Physics, University of Science and Technology of China, Hefei, Anhui 230026, P. R. China.

[3]Key Laboratory of Surface and Interface Chemistry and Energy Catalysis of Anhui Higher Education Institutes, University of Science and Technology of China, Hefei, Anhui 230026, P. R. China.

[4]Department of Chemical Physics, University of Science and Technology of China, Hefei, Anhui 230026, P. R. China.

[5]School of Chemistry & Chemical Engineering, Anhui University of Technology, Ma'anshan, Anhui 243002, P. R. China.

[†]These authors contributed equally to this work.

[*]Corresponding author. E-mail: gengzg@ustc.edu.cn (Z.G.); zengj@ustc.edu.cn (J.Z.).

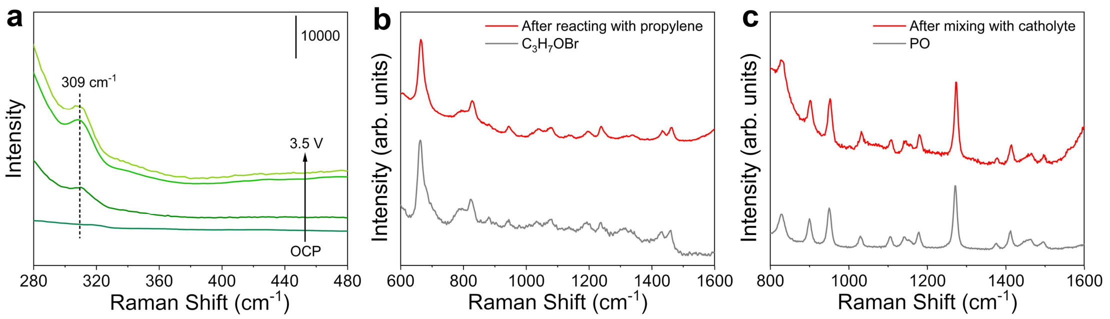

Supplementary Figure 1 | Experimental investigation of the reaction pathway. (**a**) In situ Raman spectra of KBr electrolyte at the applied potentials from open circuit to 2.9 V vs Ag/AgCl. (**b**) Raman spectra of the products after the addition reaction between HBrO and propylene. (**c**) Raman spectra of the products after the saponification process between the anolyte and catholyte. C_3H_7OBr and PO were used as the standard references, respectively. When the potential was applied, a discernible peak at 309 cm^{-1} was observed, corresponding to the Br-Br bond of Br_2. When the applied potential was further increased, the peak intensity of Br_2 was gradually enhanced, indicating the growing concentration of Br_2 in the electrolyte. Besides, after propylene was bubbled into the anolyte, the peaks at 664 and 826 cm^{-1} could be ascribed to the C-Br and C-C bonds of C_3H_7OBr, respectively. In addition, after the mixing step, the peaks at 952 and 1274 cm^{-1} were observed, corresponding to the C-O-C bond of PO.

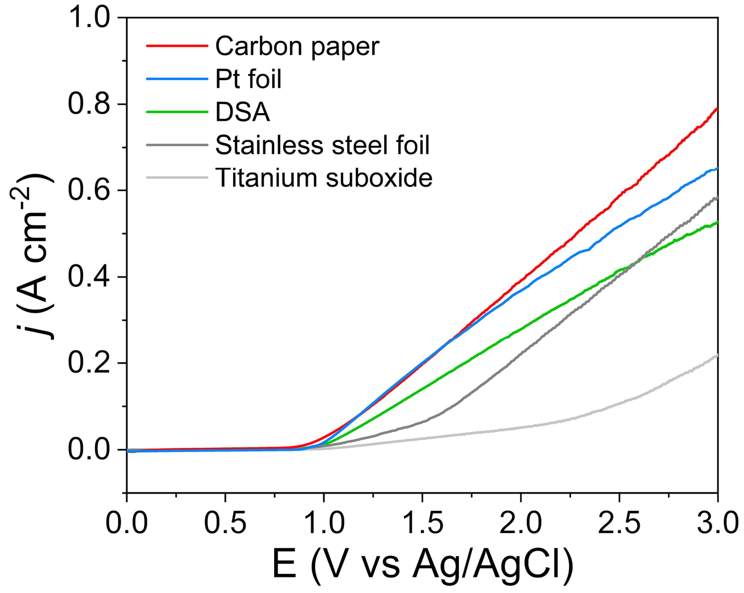

Supplementary Figure 2 | LSV measurements of various commercial electrodes including Pt foil, dimensionally stable anode (DSA), stainless steel foil, and titanium suboxide (Ti_4O_7) electrodes.

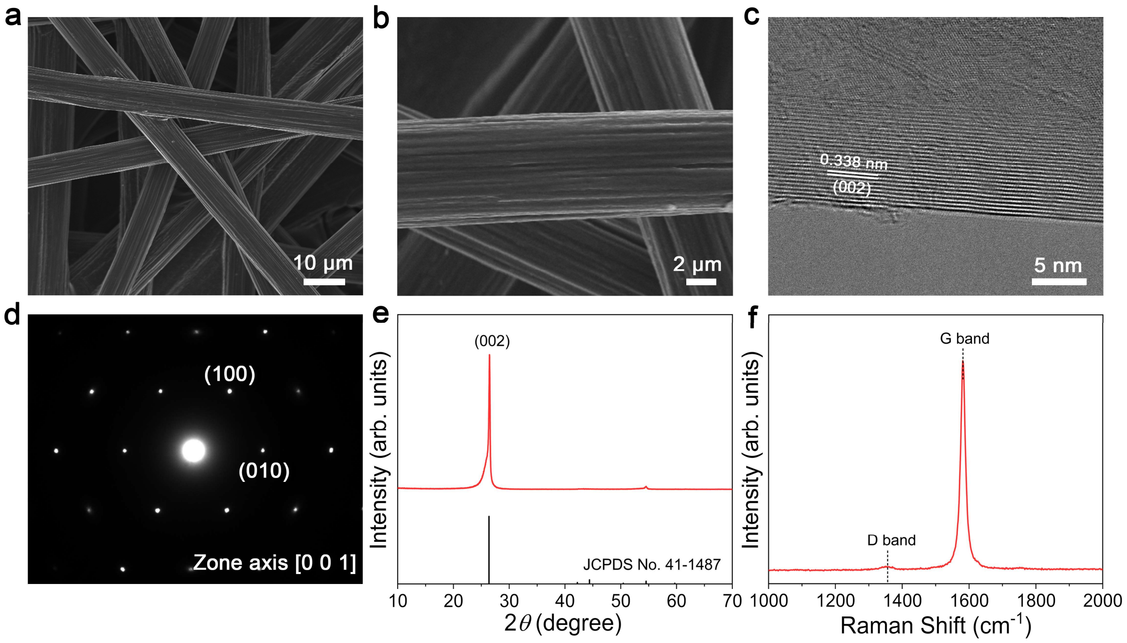

Supplementary Figure 3 | Characterization of the carbon paper. a,**b** SEM (**a**) and magnified SEM images (**b**) of the carbon paper. **c**,**d** HRTEM (**c**) and SAED images (**d**) of an individual carbon fiber. The lattice spacing of 0.338 nm was assigned to the (002) facet of graphite. **e,f** XRD pattern (**e**) and Raman spectrum (**f**) of the carbon paper. The XRD pattern matched well with the standard pattern of graphite (JCPDS No. 41-1478). The two peaks at 1356 and 1581 cm^{-1} in the Raman spectrum were assigned to the D band and G band of graphite, respectively.

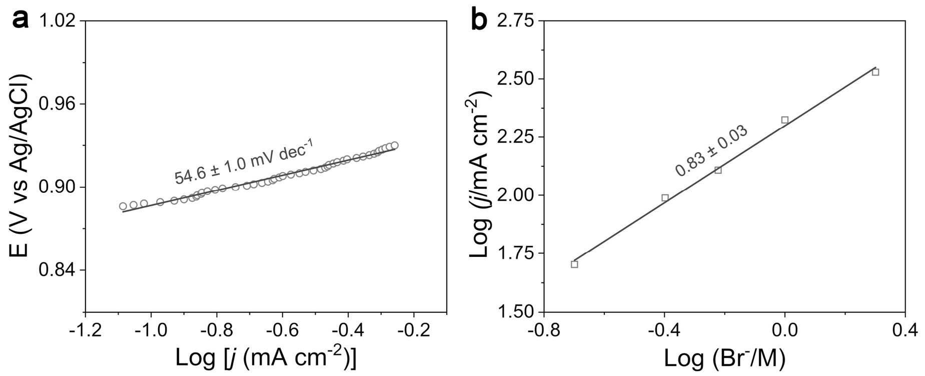

Supplementary Figure 4 | Reaction mechanism involved with Br^- electrooxidation over the carbon paper. (**a**) Tafel plot for the Br^- electrooxidation over the carbon paper. (**b**) Br^- concentration dependence of the j over the carbon paper. The Tafel slopes were 120, 40, and 30 mV dec^{-1} with the rate-determining steps of Volmer, Heyrovsky, and Tafel steps, respectively[1]. The Tafel plot of the carbon paper was 55 mV dec^{-1}, demonstrating that Br_2 was formed via the Volmer-Heyrovsky mechanism with the Heyrovsky step as the rate-determining step. Additionally, the reaction order was fitted to be 0.89, suggesting a roughly first-order dependence on Br^- concentration. Since the Heyrovsky step resembled the Eley-Rideal-type desorption involving only one Br^- adsorption, it could be concluded that the rate-determining step of Br^- electrooxidation over the carbon paper was the Heyrovsky step, which was consistent with the Tafel results[2,3].

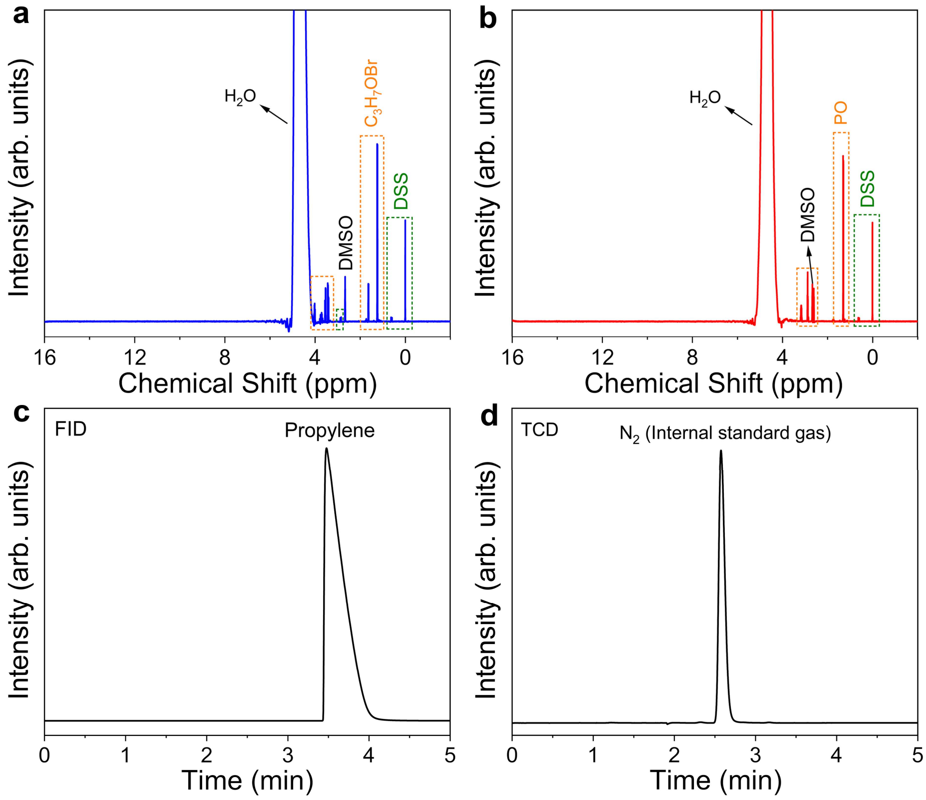

Supplementary Figure 5 | Characterization of the generated products. (**a**) ^{1}H NMR spectrum of anolyte after the addition reaction between HBrO and propylene. (**b**) ^{1}H NMR spectrum of the product after the saponification process between the anolyte and catholyte. (**c-d**) GC spectra of gaseous products. Propylene was detected by FID detector (**c**), while N_2 internal standard gas was detected by TCD detector (**d**).

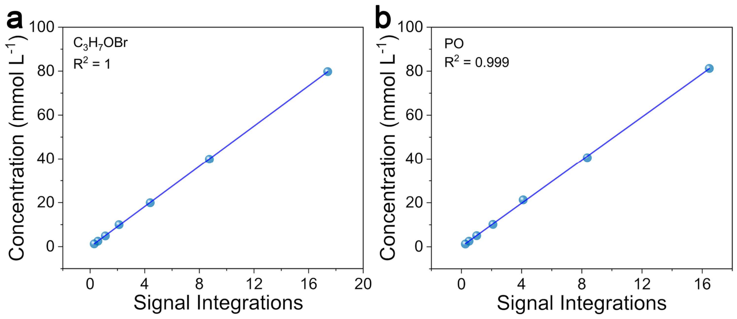

Supplementary Figure 6 | ^{1}H NMR measurements for C_3H_7OBr and PO. The corresponding concentration-integral area curves of C_3H_7OBr (**a**) and PO (**b**).

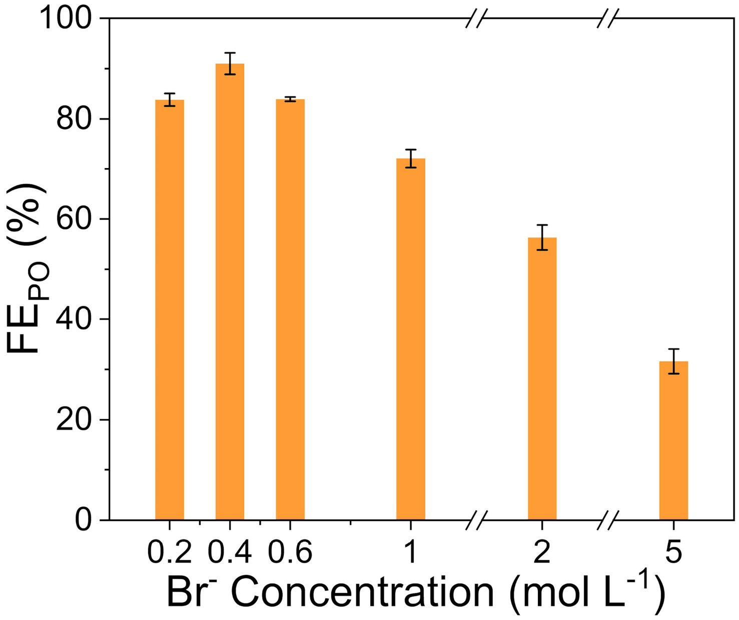

Supplementary Figure 7 | FE_{PO} in different concentrations of KBr ranging from 0.2 to 5 M at 1.9 V vs Ag/AgCl. The error bars correspond to the standard deviation of three independent measurements.

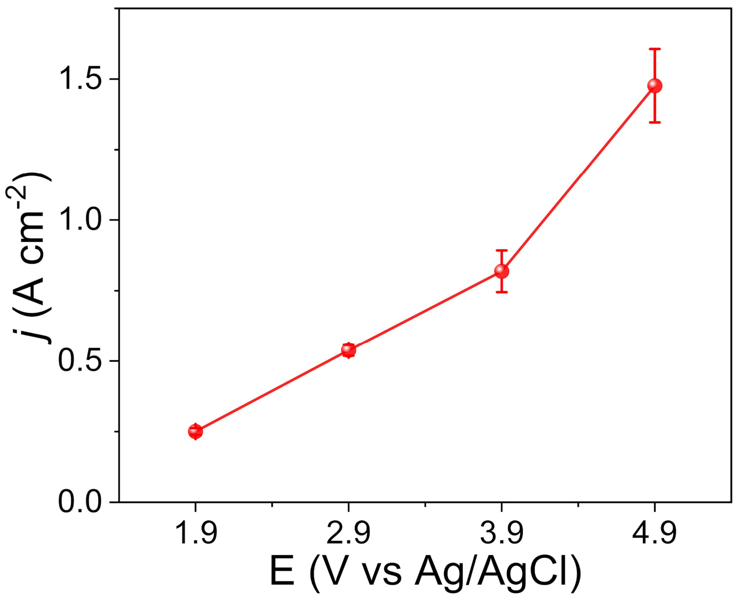

Supplementary Figure 8 | Geometrical current density at different applied potentials. The error bars correspond to the standard deviation of three independent measurements.

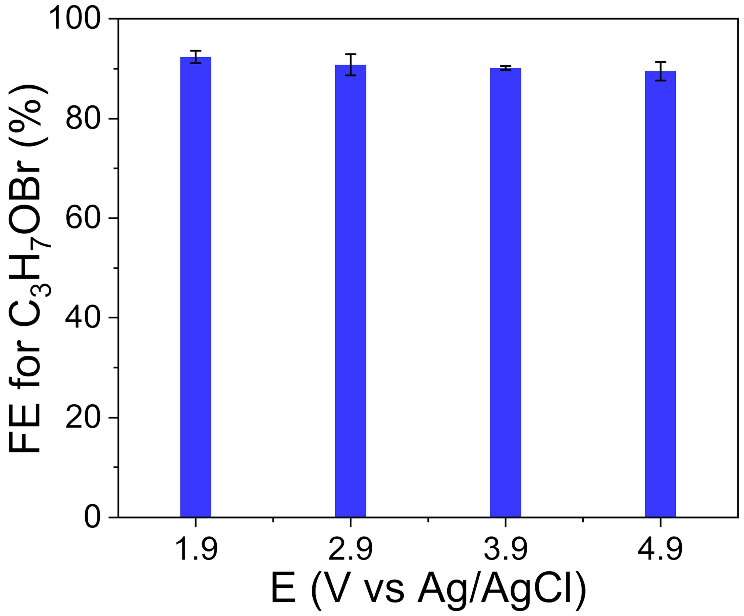

Supplementary Figure 9 | FE for C_3H_7OBr at different applied potentials. The error bars correspond to the standard deviation of three independent measurements.

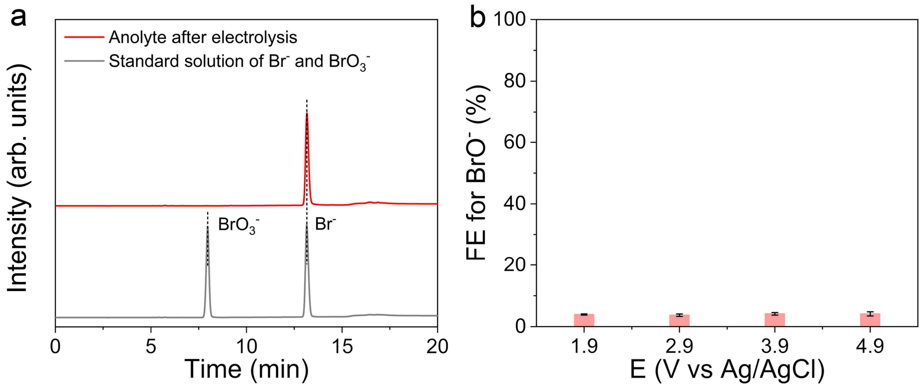

Supplementary Figure 10 | The determination of side reactions. (**a**) Profiles for BrO_3^- in the anolyte after electrolysis at 4.9 V vs Ag/AgCl determined by ion chromatography. (**b**) FE for BrO^- at different applied potentials. The error bars correspond to the standard deviation of three independent measurements.

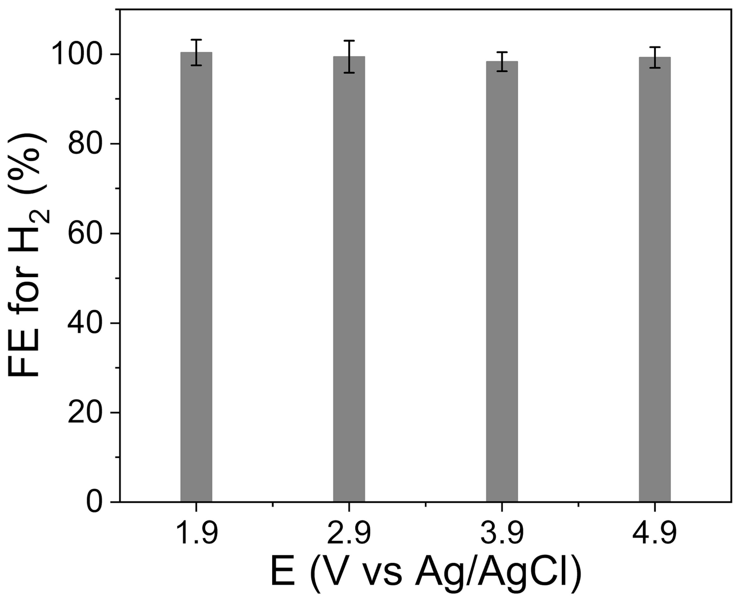

Supplementary Figure 11 | FE for H_2 at different applied potentials. The error bars correspond to the standard deviation of three independent measurements.

Supplementary Table 1 | FE_{PO} and j_{PO} at 1.9 V vs Ag/AgCl operated under different temperatures.

Temperature (°C)	FE_{PO} (%)	j_{PO} (mA cm^{-2})
20	91	237
30	91	278
40	91	315

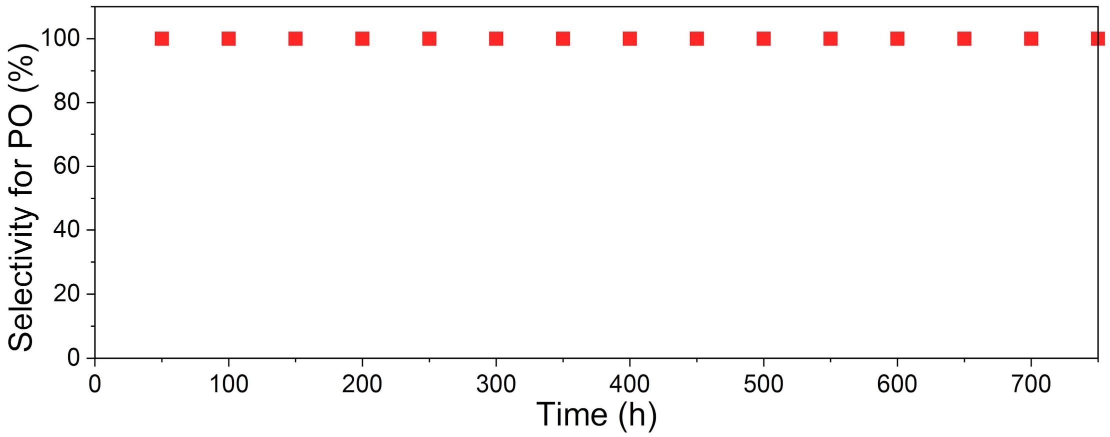

Supplementary Figure 12 | Stability test during 750-h (>30 days) continuous electrolysis at a constant current density of 250 mA cm^{-2}. The selectivity for PO was maintained above 99.9% during the continuous operation.

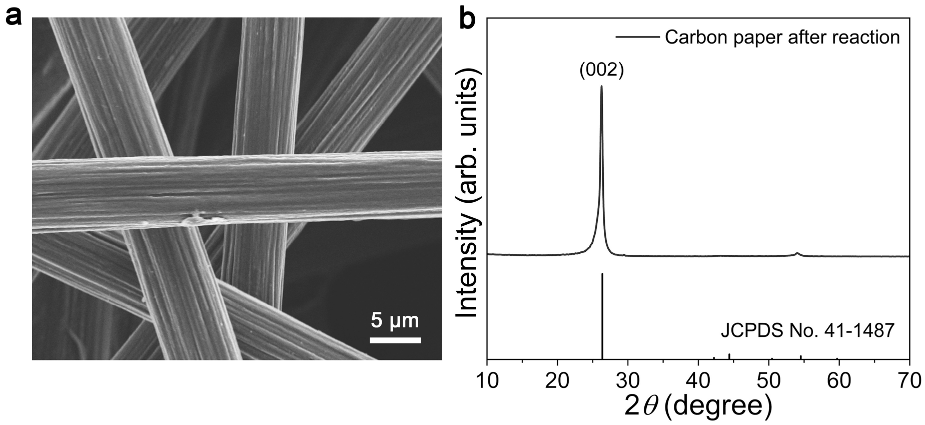

Supplementary Figure 13 | Characterization of the carbon paper after the stability test. SEM image (**a**) and XRD pattern (**b**) of the carbon paper after the 750-h electrolysis at a current density of 250 mA cm^{-2}.

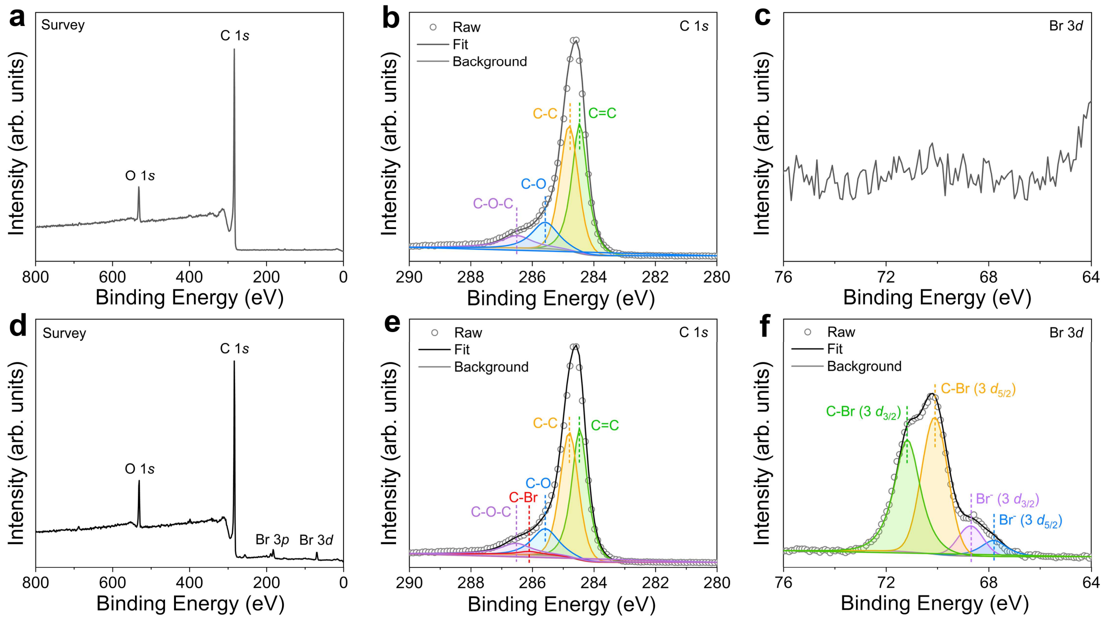

Supplementary Figure 14 | XPS spectra of the carbon paper before and after the stability test. (**a-c**) The survey (**a**), C 1*s* (**b**), and Br 3*d* (**c**) spectra of the carbon paper before the stability test. (**d-f**) The survey (**d**), C 1*s* (**e**), and Br 3*d* (**f**) spectra of the carbon paper after the stability test. For the pristine carbon paper, C 1*s* and O 1*s* peaks were observed in the survey spectra. Especially, the C 1*s* region of the pristine carbon paper was deconvoluted into four peaks at 284.5, 284.8, 285.6, and 286.5 eV, corresponding to C=C, C-C, C-O, and C-O-C bonds, respectively[4,5]. As for the carbon paper after the stability reaction, apart from the peaks of C 1*s* and O 1*s*, two new peaks ascribed to Br 3*d* and 3*p* were observed. The C 1*s* region was deconvoluted into five peaks at 284.5, 284.8, 285.6, 286.1, and 286.5 eV, respectively. The new peak located at 286.1 eV was assigned to the C-Br bonds[6]. The formation of C-Br bonds was further confirmed by the Br 3*d* spectrum. The two peaks at 70.1 and 71.2 eV corresponded to the C-Br bonds[5].

Supplementary Table 2 | C, O, and Br contents of the carbon paper during electrolysis.

Reaction time (h)	C content (%)	O content (%)	Br content (%)
0	93.2	6.8	0
0.5	90.0	8.5	1.5
10	89.8	8.6	1.6

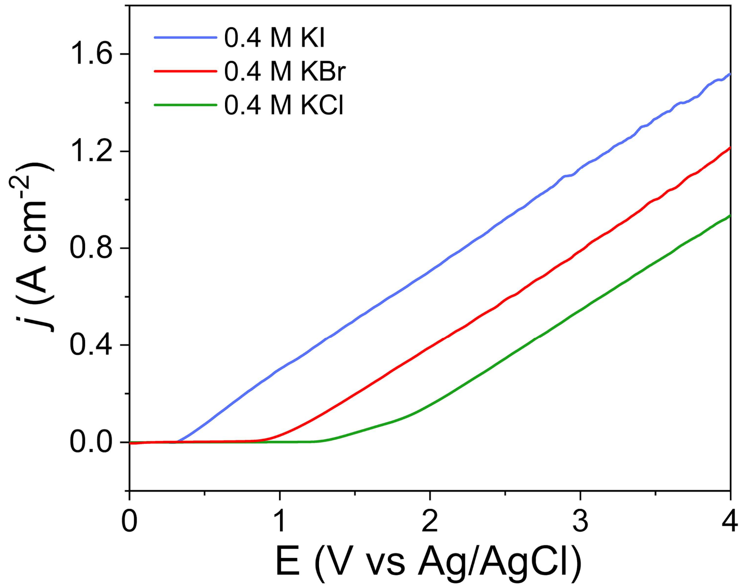

Supplementary Figure 15 | LSV curves in 0.4 M KCl, KBr, and KI ranging from 0 to 4.0 V vs Ag/AgCl, respectively.

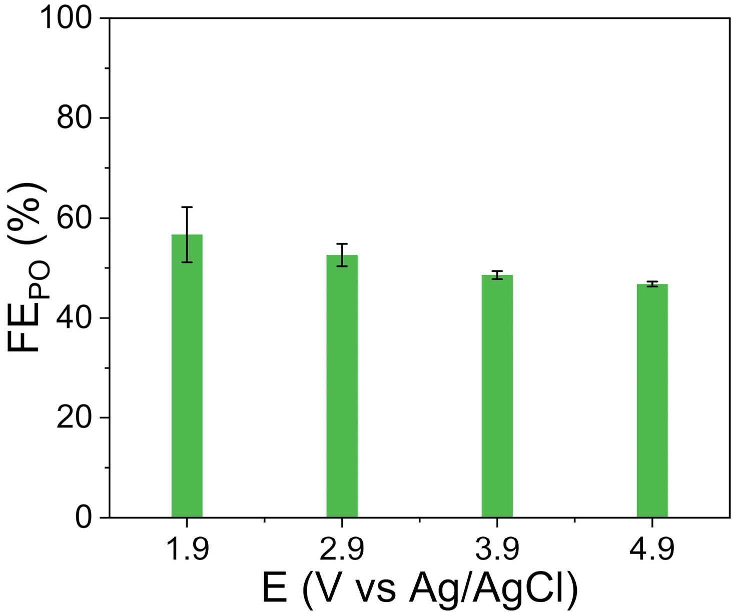

Supplementary Figure 16 | FE_{PO} at different applied potentials with 0.4 M KCl as the electrolyte. The error bars correspond to the standard deviation of three independent measurements.

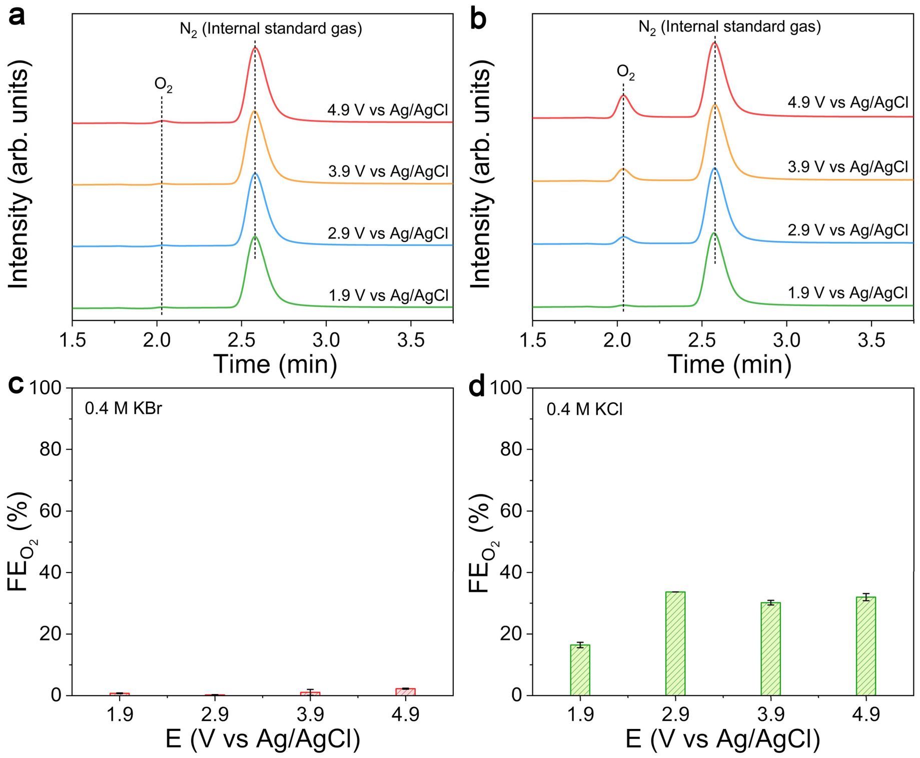

Supplementary Figure 17 | The detection of generated O_2 via the electrochemical bromohydrin and chlorohydrin routes. The GC curves of N_2 (internal standard gas) and generated O_2 with 0.4 M KBr (**a**) and KCl (**b**) as electrolytes. FE for O_2 with 0.4 M KBr (**c**) and KCl (**d**) as electrolytes. The error bars correspond to the standard deviation of three independent measurements.

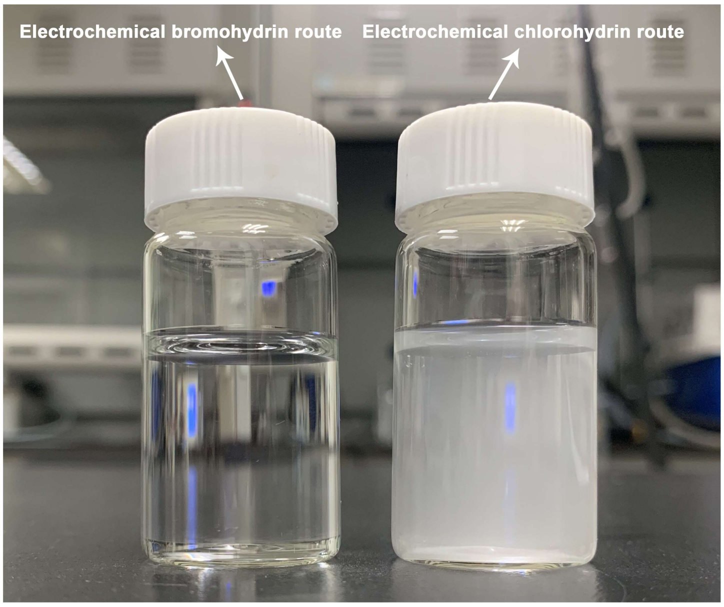

Supplementary Figure 18 | A photograph of the solutions in absorption devices after titration operations. In a typical titration operation, excess HNO_3 was added into the absorption solution to neutralize the residual NaOH, and then 0.1 M $AgNO_3$ was dripped into the above solution to determine the Br^- and Cl^-. The white precipitation of AgCl was observed in the absorption solution of Cl_2.

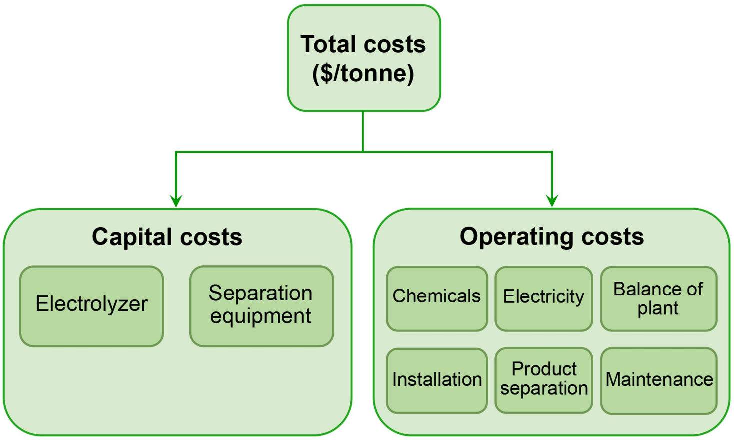

Supplementary Figure 19 | TEA analysis model for the electrochemical transformation of propylene into PO.

Techno-economic analysis (TEA)

As shown in Figure S19, to ascertain the economic potential of the electrochemical bromohydrin route, we carried out a TEA analysis model. We perform the LCP calculation taking the PO daily capacity of 10 tonnes as an example. The FE and selectivity for PO are assumed to be 90% and 98%, respectively. The current density and full cell voltage parameters of the model are all based on our experimental results. For example, at 1.9 V vs Ag/AgCl, the current density is 250 mA cm^{-2} with a cell voltage of 5.0 V. Below is a full list of the specific assumptions:

1. Based on the daily capacity of PO, the amount of propylene required is 7.4 tonnes per day.
2. The electrolyzer cost for the stack component is $5000\ \$\ m^{-2}$ according to the literature[7-9]. To calculate the capital costs, we assume that the plant has a lifetime of 10 years.
3. Capacity factor is the proportion of time that the plant is operational and this is assumed as 80%[10].
4. Separation equipment costs is assumed as 10% of the electrolyzer costs[10].
5. As for material costs, the price of propylene and PO is 1000 \$ per tonne and 1600 \$ per tonne, respectively (price data all originated from *https://www.chemanalyst.com*). The price of hydrogen is 3500 \$ per tonne. The FE for hydrogen generation is assumed as 100%.
6. The price of electricity is assumed as $0.1\ \$\ kWh^{-1}$[7-9].
7. Balance of plant factor is assumed as 35%[11].
8. Installation factor is assumed as 1.2[11].
9. Product separation costs consist of two components, distillation costs for the separation of PO and a recycle system for propylene gas. The combined cost is assumed as 20% of electricity costs[10].
10. Maintenance factor is assumed as 2.5%[11]. These costs include replenishing the catalysts and electrolytes used in the system.

Capital costs

(1) Electrolyzer costs

We first calculate the total charge required to produce 10 tonnes of PO per day.

Q = Mass of PO converted × F × N / (Molar mass of PO × FE) = 10 tonnes × 96485 × 2 / (58.08 × 90%) = 3.69×10^{10} C

Where F is the Faraday constant and N takes the value 2 since the electrochemical bromohydrin route is a two-electron transfer process.

The total current is calculated as:

I = Q / Time in a day = 3.69×10^{10} C / (24 h × 60 × 60) = 4.27×10^{5} A

The total cost of electrolyzers is determined as:

Total cost of electrolyzer = I × unit cost of electrolyzer / j = 4.27×10^{5} A × 5000 \$ m^{-2} / 250 mA cm^{-2} = 8.54×10^{5} \$

The electrolyzer cost per tonne is calculated as:

Electrolyzer cost (\$ $tonne^{-1}$) = Total cost of electrolyzer × Capital recovery factor / (Capacity factor × Time in one year × Mass of PO produced one day) = 8.54×10^{5} \$ × 0.18 / (0.8 × 365 day ×10 tonne day^{-1}) = 52.64 \$ $tonne^{-1}$

Capital recovery factor = Discount rate × (1 + Discount rate)^{Lifetime} / [(1 + Discount rate)^{Lifetime} - 1] = 12% × (1 + 12%)^{10} / [(1 + 12%)^{10} - 1] = 0.18

(2) Separation equipment costs

The liquid separation equipment costs are assumed to be 10% of electrolyzer costs:

Separation equipment costs (\$ $tonne^{-1}$) = 10% × Electrolyzer costs = 10% × 52.64 \$ $tonne^{-1}$ = 5.26 \$ $tonne^{-1}$

Therefore, the capital costs can be calculated:

Capital costs (\$ $tonne^{-1}$) = Electrolyzer costs + Separation equipment costs = 52.64 \$ $tonne^{-1}$ + 5.26 \$ $tonne^{-1}$ = 57.90 \$ $tonne^{-1}$

Operating costs

(1) Chemicals costs

Chemical costs (\$ $tonne^{-1}$) = Cost of propylene × Mass of propylene need / Mass of PO produced = 1000 \$ $tonne^{-1}$ × 7.4 tonne / 10 tonne = 740 \$ $tonne^{-1}$

(2) Electricity costs

The energy used per day can be calculated as follows:

Energy used per day (kWh) = E × I × Time in a day = 5.0 V × 4.27×10^{5} A × 24 h = 5.12×10^{7} Wh = 5.12×10^{4} kWh

The electricity cost per day normalized by the mass of propylene oxide produced can be calculated as follows:

Electricity costs (\$ $tonne^{-1}$) = Energy used per day × Cost per kWh / Mass of PO = 5.12×10^{4} kWh × 0.1 \$ kWh^{-1} / 10 tonne = 512 \$ $tonne^{-1}$

(3) Balance of plant costs

Balance of plant costs ($ $tonne^{-1}$) = Balance of plant factor × Electrolyzer costs = 35% × 52.64 $ $tonne^{-1}$ = 18.42 $ $tonne^{-1}$

(4) Installation costs

Installation costs ($ $tonne^{-1}$) = Installation factor × Capital costs = 1.2 × 57.90 $ $tonne^{-1}$ = 69.48 $ $tonne^{-1}$

(5) Product separation costs

Product separation costs ($ $tonne^{-1}$) = 20% × Electricity costs = 20% × 512 $ $tonne^{-1}$ = 102.40 $ $tonne^{-1}$

(6) Maintenance costs

Maintenance costs ($ $tonne^{-1}$) = Maintenance factor × Capital costs = 0.025 × 57.90 $ $tonne^{-1}$ = 1.45 $ $tonne^{-1}$

Therefore, the operating costs can now be calculated by adding up the above six components:

Operating costs ($ $tonne^{-1}$) = Chemical costs + Electricity costs + Balance of plant costs + Installation costs + Product separation costs + Maintenance costs = 740 + 512 + 18.42 + 69.48 + 102.4 + 1.45 = 1443.75 $ $tonne^{-1}$

Potential profit

The profit per day can be calculated based on the market price of PO and hydrogen, which are assumed to be 1600 $ $tonne^{-1}$ and 3500 $ $tonne^{-1}$, respectively. Next, we calculate the profit of hydrogen generated at the cathode with a FE of 100%.

Mass of hydrogen produced per day (tonne) = Molar mass of hydrogen × Q / (N × F) = 3.69×10^{10} C × 2 g mol^{-1} / 2 × 96485 C mol^{-1} = 0.38 tonne

Profit from hydrogen can now be normalized based on the 10 tonnes of PO produced per day:

Profit from hydrogen ($ $tonne^{-1}$) = Price of hydrogen × Mass of hydrogen produced per day / Mass of PO = 3500 $ $tonne^{-1}$ × 0.38 tonne / 10 tonne = 133 $ $tonne^{-1}$

Therefore, total profits per tonne of PO:

Total profit per tonne of PO produced ($ $tonne^{-1}$) = Profit from hydrogen + 1700 - Capital costs - Operating costs = 133 + 1600 - 57.90 - 1443.75 = 231.35 $ $tonne^{-1}$

Calculation of NPV

NPV is estimated by calculating the yearly cash flows over the lifetime of the facility[11]. The total capital expenditure is given by the sum of capital costs. The total income tax is assumed as 38.9% and the nominal interest rate is 10%. The working capital is 5% of the capital costs. Here we perform an example NPV calculation for the case where the operating current density is assumed as 250 mA cm^{-2}.

In year zero, the facility is built, leading to a cumulative present value of:

$$-\$8.54 \times 10^5 \times 1.1 = -\$939400$$

$$\text{Cumulative value of Year 0} = -\$939400 - \$939400 \times 5\% = -\$986370$$

In year 1, the cash flow is calculated as follows:

$$\text{Depreciation of Year 1} = \$939400 \times 10\% = \$93940$$

$$\text{Net earnings of Year 1} = (\$230.85 \times 10 \times 365 \times 0.8 + \$93940) \times (1 - 0.389) = \$469261$$

$$\text{Discounted cash flow of Year 1} = \$469261 - \$93940 = \$375321$$

$$\text{Cash flow of Year 1} = \$375321 \times 1 / (1+0.1)^1 = \$341201$$

$$\text{Cumulative present value of Year 1} = -\$986370 + \$341201 = -\$645169$$

These calculations are repeated each year during the lifetime of the facility to provide the end-of-life NPV.

Supplementary Table 3 | Range of values for sensitivity analysis.

Parameters	Better	Base	Worse
Cell voltage (V)	2.8	5.0	11.8
j (A/cm^2)	1.5	0.25	0.038
FE_{PO} (%)	100	90	40
Electrolyzer cost ($/m^2)	1000	5000	8000
Propylene cost ($/tonne)	800	1000	1200
Renewable electricity cost (¢/kWh)	5	10	15

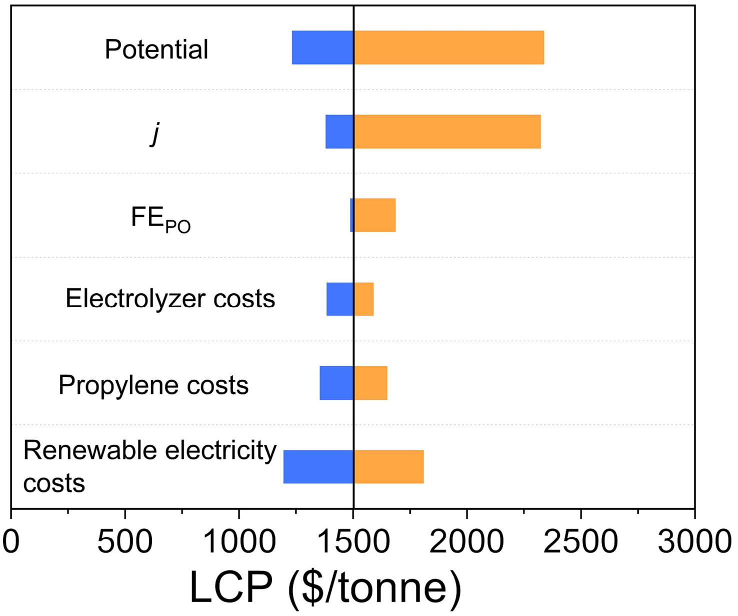

Supplementary Figure 20 | Sensitivity analysis of the LCP for PO production.

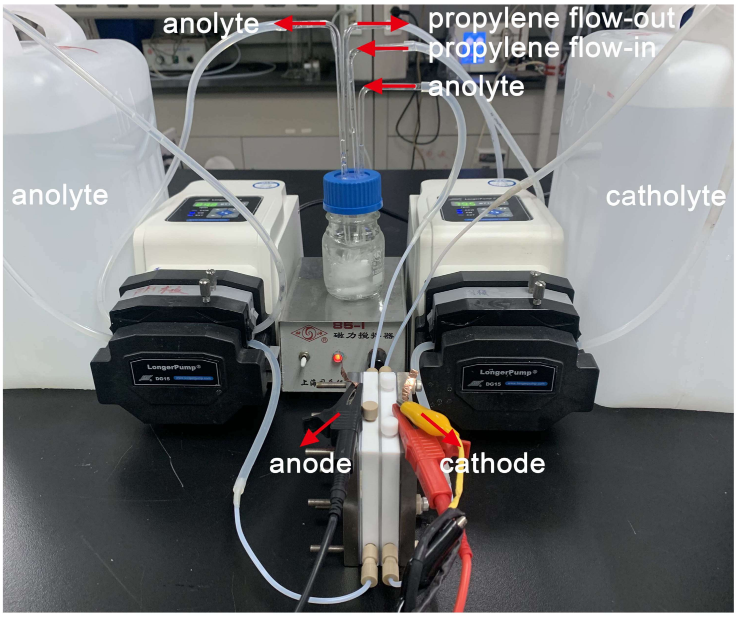

Supplementary Figure 21 | A photograph of the customized testing device.

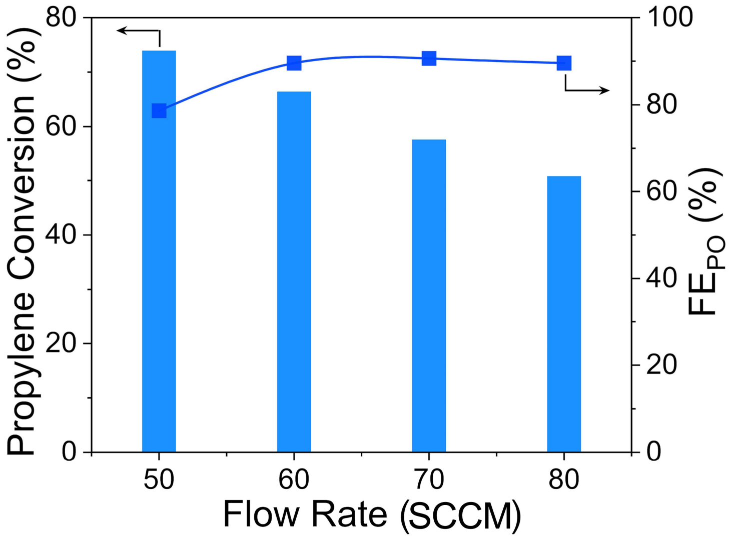

Supplementary Figure 22 | The single-pass conversion of propylene and FE_{PO} at different flow rates of propylene.

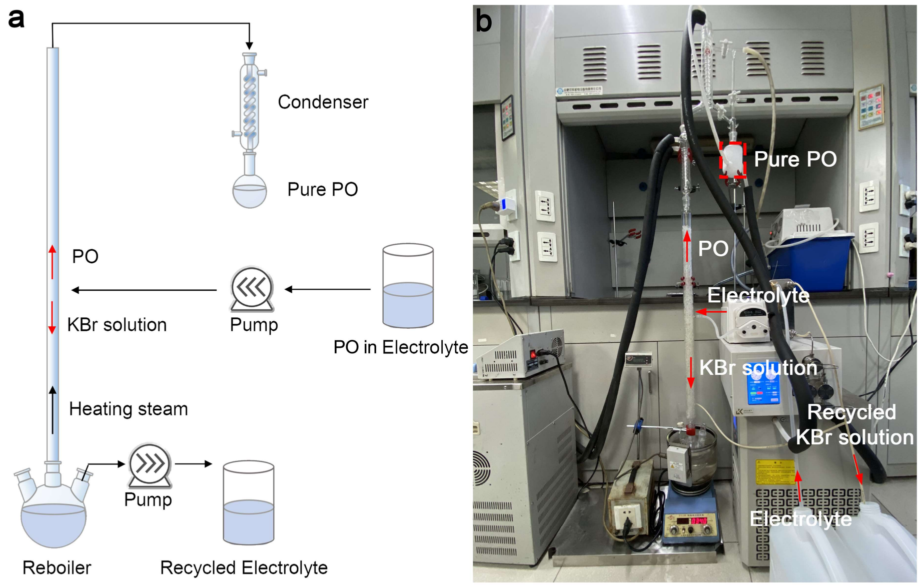

Supplementary Figure 23 | Designed vacuum distillation unit for PO. (**a**) Schematic of the designed vacuum distillation unit for PO. (**b**) A photograph of the designed vacuum distillation unit for PO.

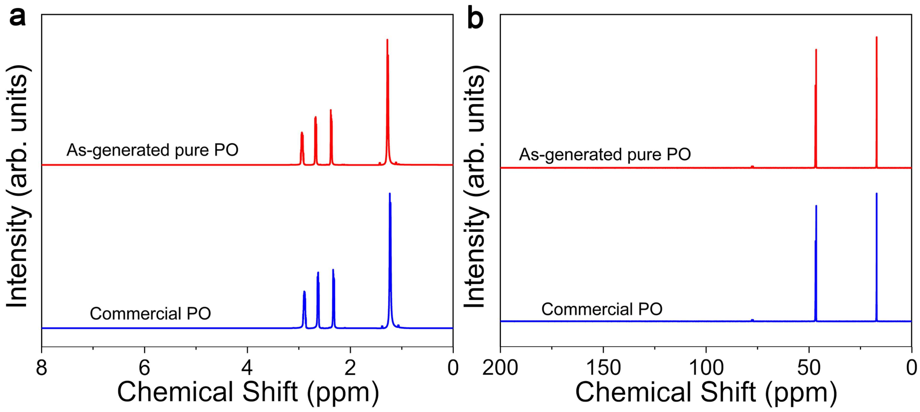

Supplementary Figure 24 | NMR spectra of the pure PO generated from the designed vacuum distillation unit. Typical ^{1}H (**a**) and ^{13}C (**b**) NMR spectra of the pure PO generated from the designed vacuum distillation unit, demonstrating that no other impurity was formed compared with the commercial PO.

References

S1. Shinagawa, T., Garcia-Esparza, A. T. & Takanabe, K. Insight on tafel slopes from a microkinetic analysis of aqueous electrocatalysis for energy conversion. *Sci Rep* **5**, 13801 (2015).

S2. Tilak, B. V. & Conway, B. E. Analytical relations between reaction order and tafel slope derivatives for electrocatalytic reactions involving chemisorbed intermediates. *Electrochim. Acta* **37**, 51-63 (1992).

S3. Lim, T., Kim, J. & Joo, S. H. Electrocatalysis of selective chlorine evolution reaction: fundamental understanding and catalyst design. *J. Electrochem. Sci. Technol.* **14**, 105-119 (2023).

S4. Lv, Q. et al. Selectively nitrogen-doped carbon materials as superior metal-free catalysts for oxygen reduction. *Nat. Commun.* **9**, 3376 (2018).

S5. Rabchinskii, M. K. et al. From graphene oxide towards aminated graphene: facile synthesis, its structure and electronic properties. *Sci Rep* **10**, 6902 (2020).

S6. Chen, X., Wang, X. & Fang, D. A review on C 1*s* XPS-spectra for some kinds of carbon materials. *Fuller. Nanotub. Carbon Nanostruct.* **28**, 1048-1058.

S7. Gao, Y. et al. Membrane-free electrosynthesis of epichlorohydrins mediated by bromine radicals over nanotips. *J. Am. Chem. Soc.* **146**, 714-722 (2024).

S8. Zhao, B. H. et al. Economically viable electrocatalytic ethylene production with high yield and selectivity. *Nat. Sustain.* **6**, 827-837 (2023).

S9. Shin, H., Hansen, K. U. & Jiao, F. Techno-economic assessment of low-temperature carbon dioxide electrolysis. *Nat. Sustain.* **4**, 911-919 (2021).

S10. Lum, Y. et al. Tuning OH binding energy enables selective electrochemical oxidation of ethylene to ethylene glycol. *Nat. Catal.* **3**, 14-22 (2020).

S11. Jouny, M., Luc, W. & Jiao, F. General techno-economic analysis of CO_2 electrolysis systems. *Ind. Eng. Chem. Res.* **57**, 2165-2177 (2018).
